# Synergistic Adsorption by Easily Retrievable Magnetic MOF Composites for Enhanced Removal of Benzoic Acid from Water

**DOI:** 10.3390/molecules31162759

**Published:** 2026-08-08

**Authors:** Panpan Liu, Peijun He, Yixin Wu, Xiufang Li, Xianmang Xu, Jianing Duan, Peng Bai

**Affiliations:** 1 College of Pharmacy, Heze University, Heze 274015, China; 2 Heze Branch, Qilu University of Technology (Shandong Academy of Sciences), Heze 274000, China; peijun_he2026@163.com (P.H.); wuyixin0105@163.com (Y.W.); xuxianmang168@qlu.edu.cn (X.X.); djn@qlu.edu.cn (J.D.); 3 Department of Pharmaceutical Engineering, School of Chemical Engineering and Technology, Tianjin University, Tianjin 300350, China; baipenga525@tju.edu.cn; 4 Key Laboratory of Systems Bioengineering, Ministry of Education, Tianjin 300350, China

**Keywords:** magnetic UiO-66, synergistic efficacy, adsorption, benzoic acid, mechanism

## Abstract

In this study, we prepared a magnetic University of Oslo-66 (UiO-66) composite material, Fe_3_O_4_@SiO_2_@UiO-66, and applied it as an adsorbent for the effective removal of benzoic acid from wastewater. The resulting magnetic MOF composite retained the structural and property characteristics of MOFs, along with magnetic separation capabilities, allowing for swift recovery from water through external magnetic fields. A potential synergistic effect between magnetic particles and UiO-66 was observed, leading to enhanced benzoic acid adsorption. The adsorption capacity per Zr_6_ cluster unit in Fe_3_O_4_@SiO_2_@UiO-66 measured 569.5 g/mol, surpassing that of UiO-66 by more than 26%. We conducted investigations into adsorption properties as a function of initial concentration, contact time, and pH values. The adsorption of magnetic UiO-66 composites was well described by pseudo-second-order and Langmuir models, indicating predominant chemisorption on the homogeneous surface. Thermodynamic studies of adsorption revealed a spontaneous exothermic process. Furthermore, we analyzed possible mechanisms of benzoic acid adsorption, highlighting the contributions of electrostatic interactions, coordination interactions, and π–π stacking interactions. Magnetic UiO-66 exhibited remarkable adsorption capacity, along with reliable regeneration performance and convenient magnetic separation capabilities, making it an exceptional adsorbent for industrial applications in the removal of benzoic acid.

## 1. Introduction

A multitude of industrial plants, including the petrochemical, paper, leather, textile, and plastics industries, produce large amounts of wastewater containing cyclic organic compounds, which have a significant impact on water quality and are very harmful to human health and ecosystems [1,2,3]. These wastewaters contain a very common cyclic organic compound, benzoic acid (BA), which is widely used as a major reaction intermediate or chemical preservative [1,4,5,6]. At the same time, BA will inevitably accumulate in wastewater during its production and application. As a result, wastewaters containing BA must undergo treatment before discharging into receiving waterbodies. Currently, removing and recycling BA from wastewater remains a massive challenge [7,8].

To date, a number of physical, biological, and chemical treatments have been reported for removing BA from aqueous solutions [9,10,11,12,13]. Among them, adsorption has been proven to be an effective method for treating wastewater containing BA, because of its simplicity, efficiency, and energy-saving qualities, as well as its non-destructive nature [4,13,14]. A variety of adsorbents, such as clay minerals, synthesized polymers, activated carbon, and mesoporous materials, have been successfully applied to remove BA from water and achieved favorable separation results [15,16,17,18,19]. Despite their promise for wastewater treatment, these materials were difficult to prepare, had poorly controlled structures, and possessed inadequate surface areas and insufficient active surface sites, which hindered their excellent adsorption properties and sustainability. Therefore, to qualify as an exceptional adsorbent, it must possess a substantial specific surface area, a profusion of high-affinity adsorption sites, and ease of separation capabilities. Consequently, it should offer swift mass-transfer channels, ample adsorption capacity, and abundant adsorption sites, and it should exhibit superior performance in both adsorption and separation processes, all of which collectively enable the rapid and efficient removal of BA.

Metal–organic frameworks (MOFs) represent a novel and intriguing class of microporous materials, comprising metal clusters intricately connected by organic ligands [2,20,21]. Owing to their high specific surface areas, designable pore sizes and shapes, tunable functional groups, and abundant active metal sites, MOFs hold tremendous potential for the adsorption and treatment of water pollutants [22,23,24]. As a typical MOF material, UiO-66 is composed of Zr_6_ cluster [Zr_6_O_4_(OH)_4_] and terephthalate linkers [25,26], which has high thermal and chemical stability [27,28] and excellent adsorption performances for removing pollutants in aqueous solution, such as heavy metal ions, organic dyes, and antibiotics [29,30,31]. UiO-66 is particularly promising for the extraction of BA from wastewater for several reasons. First, the strong Zr–O coordination bonds endow UiO-66 with exceptional stability in water and acidic media [32,33], making it particularly suitable for the treatment of acidic contaminants such as BA. Second, the aromatic terephthalate linkers can interact with the benzene ring of BA through π–π stacking interactions, as reported for the adsorption of other aromatic acids such as salicylic acid on UiO-66 [34,35,36]. Third, the Zr_6_ clusters and their defect-associated open Zr sites provide Lewis acid sites capable of coordinating the carboxylate group of BA, similar to the adsorption of p-arsanilic acid and fluoride on UiO-66 [37,38]. Finally, the pore apertures of UiO-66 (ca. 6 Å) match the molecular dimensions of BA, allowing rapid mass transfer into the cages [39], and MOF-based materials have been proven effective for the removal of BA and structurally related aromatic acids from water [1,4]. These features make UiO-66 an attractive candidate for extracting BA from wastewater. Nevertheless, UiO-66 is typically obtained as a microcrystalline powder, which is difficult to separate and recover from aqueous solutions after use, posing a risk of secondary pollution and limiting its industrial application. To address this issue, researchers have attempted to combine membranes with MOFs [40]. However, firmly immobilizing MOF particles onto a substrate membrane remains a significant challenge due to the weak interfacial bonding between them.

Magnetic separation technology is an effective method for recovering powder adsorbents from solutions, thus expanding the applications of adsorbents in engineering separation [41,42]. Furthermore, magnetic nanoparticles have demonstrated efficacy in removing various common inorganic and organic pollutants [43,44,45,46]. By incorporating magnetic particles into MOFs, the resulting composite inherits the excellent adsorption performance of MOFs and the advantages of magnetic materials for magnetic separation. As a result, the adsorption process can be simplified by avoiding tedious centrifugation or filtration procedures, making it a major focus of research in the field of adsorption separation. It is worth noting that the combination of magnetic particles and UiO-66 is expected to produce a synergistic effect rather than a simple superposition of the two components: (i) the silanol groups on the silica surface can template the heterogeneous nucleation of a more complete UiO-66 framework with fewer structural (missing-linker) defects, providing more intact Zr–O coordination environments per Zr_6_ cluster; (ii) the anchoring of the MOF on the magnetic core suppresses the aggregation of UiO-66 nanocrystals and improves the accessibility of the adsorption sites. Consequently, the adsorption capacity normalized to the Zr_6_ clusters of the composite is expected to exceed that of pristine UiO-66, and the composite can still be rapidly retrieved from water by an external magnetic field.

This study successfully synthesized a magnetic UiO-66 composite and employed it for the first time in the effective removal of benzoic acid (BA) from wastewater. The synthesized materials underwent comprehensive characterization, and their adsorption properties were exhaustively explored, accompanied by the proposal of the underlying adsorption mechanism. Furthermore, the study investigated the regeneration and reusability of the adsorbent. The findings from this research establish a straightforward and highly efficient adsorption method for the removal of BA from wastewater.

## 2. Experimental Section

### 2.1. Materials

The reagents used in this research were employed without any additional purification. Zirconium (IV) chloride (ZrCl_4_, 99.9%) and benzoic acid (BA, 99.5%) were procured from Aladdin in Shanghai, China. Terephthalic acid (H_2_BDC, 99.0%), tetraethyl orthosilicate (TEOS, 98.0%), and Fe_3_O_4_ magnetic particles (99.5%) were sourced from Macklin in Shanghai, China. N, N-dimethylformamide (DMF) and ethanol were obtained from Damao in Tianjin, China. Deionized water was employed throughout the study.

### 2.2. Synthesis of Magnetic UiO-66

First, the core–shell structure was achieved by TEOS coating Fe_3_O_4_ particles, resulting in the formation of Fe_3_O_4_@SiO_2_ magnetic particles [47]. Magnetic UiO-66 was then prepared in accordance with prior research, with slight adjustments [48]. Specifically, we added 0.233 g of ZrCl_4_ and 0.1 g Fe_3_O_4_@SiO_2_ magnetic particles to a 20 mL DMF solution and mixed ultrasonically for 30 min. Subsequently, an additional 20 mL of DMF solution containing 0.166 g of H_2_BDC was added, and the mixture was stirred until complete dissolution. The two solutions were combined in a 100 mL flask and mechanically stirred at 120 °C for 24 h. A magnetic field was used to separate the product, then three wash cycles with DMF and anhydrous ethanol, respectively, were performed to obtain Fe_3_O_4_@SiO_2_@UiO-66. Finally, the sample was dried in a vacuum oven at 60 °C for 12 h and stored for subsequent use. In comparison, the same processes were also used to prepare the UiO-66 without Fe_3_O_4_@SiO_2_.

### 2.3. Characterization

Various characterization techniques, including scanning electron microscopy (SEM), transmission electron microscopy (TEM), X-ray diffraction (XRD), N_2_ adsorption measurements, X-ray photoelectron spectroscopy (XPS), thermal gravimetric analysis (TGA), Fourier-transform infrared spectroscopy (FTIR), vibrating sample magnetometry (VSM), and zeta potential measurements, were used for characterizing the synthesized materials. Sample morphology was assessed through scanning electron microscopy (SEM) using a Hitachi S-4800 instrument and transmission electron microscopy (TEM) analysis conducted with a Jeol Jem-2100F microscope. Elemental analysis was carried out through energy-dispersive spectroscopy (EDS), which was integrated with TEM. X-ray diffraction (XRD) patterns were acquired employing a D/MAX-2500 X-ray Powder Diffractometer equipped with a Cu sealed tube, covering the range of 5–50° at a temperature of 298 K. The FTIR spectra were obtained on a Nicolet 6700 spectrophotometer, with a data acquisition range of 4000–400 cm^−1^ and a resolution of 4 cm^−1^, using KBr pellets. N_2_ adsorption and desorption isotherms of the prepared materials were conducted at −196 °C using a Micromeritics ASAP2460 surface area analyzer. Thermal gravimetric analysis (TGA) data were obtained using a STA449-F3 (NETZSCH) thermogravimetric analyzer with a heating rate of 5 °C/min in an air atmosphere. XPS analyses were conducted using a PHI 1600 ESCA instrument provided by PerkinElmer (Waltham, MA, USA), equipped with Al Kα X-ray radiation. Zeta potentials at various pH values were measured with a Mastersizer 3000 instrument (Malvern Panalytical, Malvern, UK). Additionally, the magnetic properties of the nanoparticles were investigated utilizing a vibrating sample magnetometer (VSM, SQUID-VSM, Quantum Design, San Diego, CA, USA). Data processing and graph plotting were performed using Origin 2026 (OriginLab Corporation, Northampton, MA, USA).

### 2.4. Adsorption Studies

For the removal of water molecules or solvents, the synthetic samples were dried in a vacuum oven for 10 h at 60 °C before BA adsorption. Adsorption experiments are typically performed by adding exactly 20 mg of adsorbent into a solution of known concentration (20 mL) of BA aqueous solution. An incubator at a specific temperature was used to shake the mixture for 4 h, after which Fe_3_O_4_@SiO_2_@UiO-66 was separated with a magnet. The quantification of BA concentrations before and after adsorption was performed using high-performance liquid chromatography (HPLC) on an UltiMate3000 instrument (Thermo Fisher Scientific, Waltham, MA, USA), equipped with a UV detector set at a wavelength of 230 nm. A C18 column measuring 250 mm in length was employed. The mobile phase consisted of a mixture of 5% (*v*/*v*) methanol and 95% ammonium acetate aqueous solution with a concentration of 20 mmol/L [49]. The adsorption experiments were conducted in triplicate, and the data are presented as mean ± standard deviation.

### 2.5. Adsorption Kinetics Experiments

To investigate the adsorption kinetics, 20 mg of magnetic UiO-66 was introduced into 20 mL of benzoic acid (BA) aqueous solutions with initial concentrations of 500, 1000, and 2000 mg/L, respectively. The mixtures were then agitated at 30 °C in 50 mL plastic flasks, and samples of the supernatants were periodically collected during the adsorption process. These collected mixtures were separated using a magnet, and the remaining BA concentrations in the solutions were determined using High-Performance Liquid Chromatography (HPLC) to study the kinetic adsorption behavior.

To delve deeper into the adsorption kinetics of Fe_3_O_4_@SiO_2_@UiO-66, two well-established models, namely the pseudo-second-order model and the intraparticle diffusion model, were used to elucidate the kinetics of the adsorption process [50]. The pseudo-second-order model is predicated on the assumption of chemisorption, whereas the intraparticle diffusion model serves to investigate the rate-controlling step within the adsorption process. The linear shapes of these two models are, respectively, expressed as follows:

(1) $$\frac{t}{Q_{t}}=\frac{1}{k_{2}Q_{e}^{2}}+\frac{1}{Q_{e}}t$$

(2) $$Q_{t}=k_{i}t^{0.5}+K$$

where *Q_t_* and *Q_e_* (mg·g^−1^) represent the adsorption capacity at time t (h) and at equilibrium, respectively; *k*_2_ (g·mg^−1^·h^−1^) and *k_i_* (mg·g^−1^·h^−0.5^) are the rate constants of pseudo-second-order and intraparticle diffusion, respectively. The constant *K* (mg·g^−1^) is related to the boundary layer thickness: a *K* value of zero indicates that the plot of *Q_t_* versus *t*^0.5^ passes through the origin and that the adsorption process is controlled solely by intraparticle diffusion, whereas a non-zero *K* value implies that film (boundary layer) diffusion also contributes to the rate-controlling steps.

### 2.6. Isotherms and Thermodynamics of Adsorption

To investigate the adsorption isotherms and thermodynamics, a series of adsorption experiments were conducted at various temperatures (10 °C, 30 °C, and 50 °C) using different concentrations of BA solutions (500, 1000, 1500, 2000, and 3000 mg/L). In each experiment, 1 g/L of the adsorbent was added to the respective batch of BA solutions. These mixtures were then placed in an incubator and shaken for 4 h. Subsequently, the magnetic adsorbents were separated from the solution using a magnet, and the residual BA concentrations in the solutions were determined through HPLC.

To examine adsorption behaviors, three models were applied: Langmuir, Freundlich, and Henry isotherms [51,52]. The Langmuir model characterizes adsorption as taking place on a monolayer surface with no interaction between adsorbate molecules. The Freundlich adsorption model, being an empirical formulation, was utilized to describe the adsorption of multiple layers on heterogeneous surfaces. The Henry model, on the other hand, is an idealized model that illustrates the linear relationship between BA concentration and adsorption capacity. The linear representations of these three models can be expressed as follows:

(3) $$\frac{C_{e}}{Q_{e}}=\frac{1}{k_{A}Q_{m}}+\frac{1}{Q_{m}}C_{e}$$

(4) $$\log Q_{e}=\log K_{F}+\frac{1}{n}\log C_{e}$$

(5) $$Q_{e}=K_{p}C_{e}$$

where *Q_e_* (mg·g^−1^) and *C_e_* (mg·L^−1^) represent the adsorption capacity of BA and the concentration of the remaining BA solution at equilibrium, respectively; *Q_m_* (mg·g^−1^) denotes the theoretical maximum adsorption capacity of the adsorbent, attainable when BA molecules form a uniform monolayer on the adsorbent’s surface; *K_A_* (L·mg^−1^) represents the Langmuir adsorption equilibrium constant, intricately tied to the free adsorption energy. *K_F_* (mg/g(L/mg)1/n) represents the Freundlich constant, while 1/*n* (unitless) characterizes the adsorption intensity. *K_P_* (mL·g^−1^) is indicative of the distribution constant.

The thermodynamic parameters governing the adsorption of BA on Fe_3_O_4_@SiO_2_@UiO-66, encompassing the Gibbs free energy change (Δ*G*), entropy change (Δ*S*), and enthalpy change (Δ*H*), were determined using the following equations [53]:

(6) $$\ln K_{p}=\frac{\Delta S}{R}-\frac{\Delta H}{RT}$$

(7) $$\Delta G=-RT\ln K_{p}$$

where *R* represents the universal gas constant, equal to 8.314 J·mol^−1^·K^−1^; *K_p_* stands for the distribution coefficient; and *T* denotes the absolute temperature in Kelvin (K). According to Equation (6), the values of Δ*H* and Δ*S* could be obtained from the slope (−Δ*H*/*R*) and the intercept (Δ*S*/*R*) of the van’t Hoff plot (ln*K_p_* versus 1/*T*), respectively, while Δ*G* was calculated using Equation (7).

### 2.7. Regeneration and Recycling

After BA adsorption, Fe_3_O_4_@SiO_2_@UiO-66 (0.2 g) was collected and regenerated by sonication with 100 mL ethanol for 4 h, followed by oscillation at 50 °C for 24 h. Subsequently, the sample underwent multiple wash cycles with water and ethanol before being dried for 10 h under vacuum conditions at 60 °C. The regenerated materials were then employed for subsequent adsorption–desorption cycles and subjected to XRD analysis.

## 3. Results and Discussion

### 3.1. Characterization of the Sample

**Figure 1:**
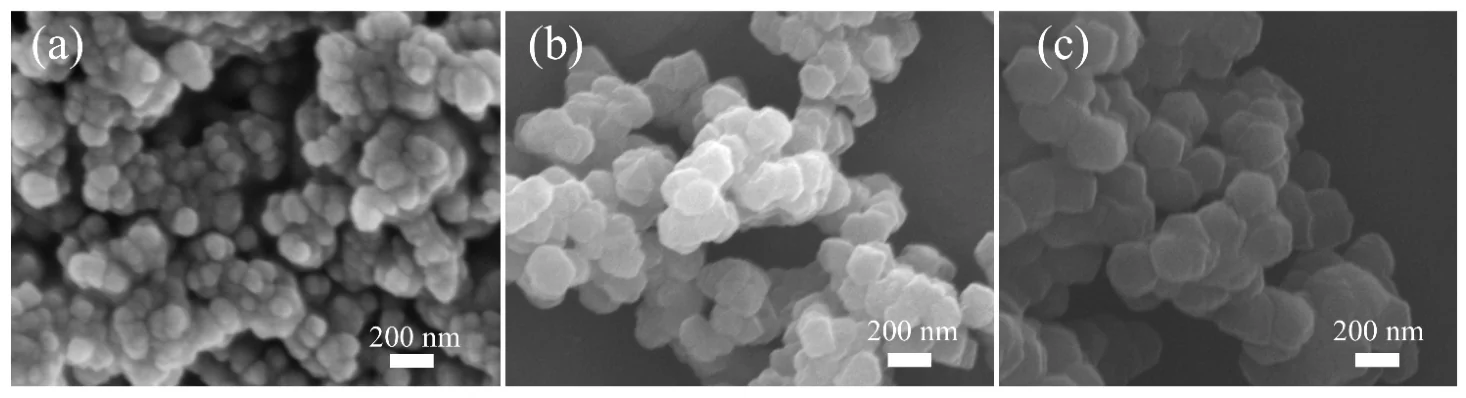
SEM images of (**a**) Fe_3_O_4_, (**b**) Fe_3_O_4_@SiO_2_, and (**c**) Fe_3_O_4_@SiO_2_@UiO-66.

**Figure 2:**
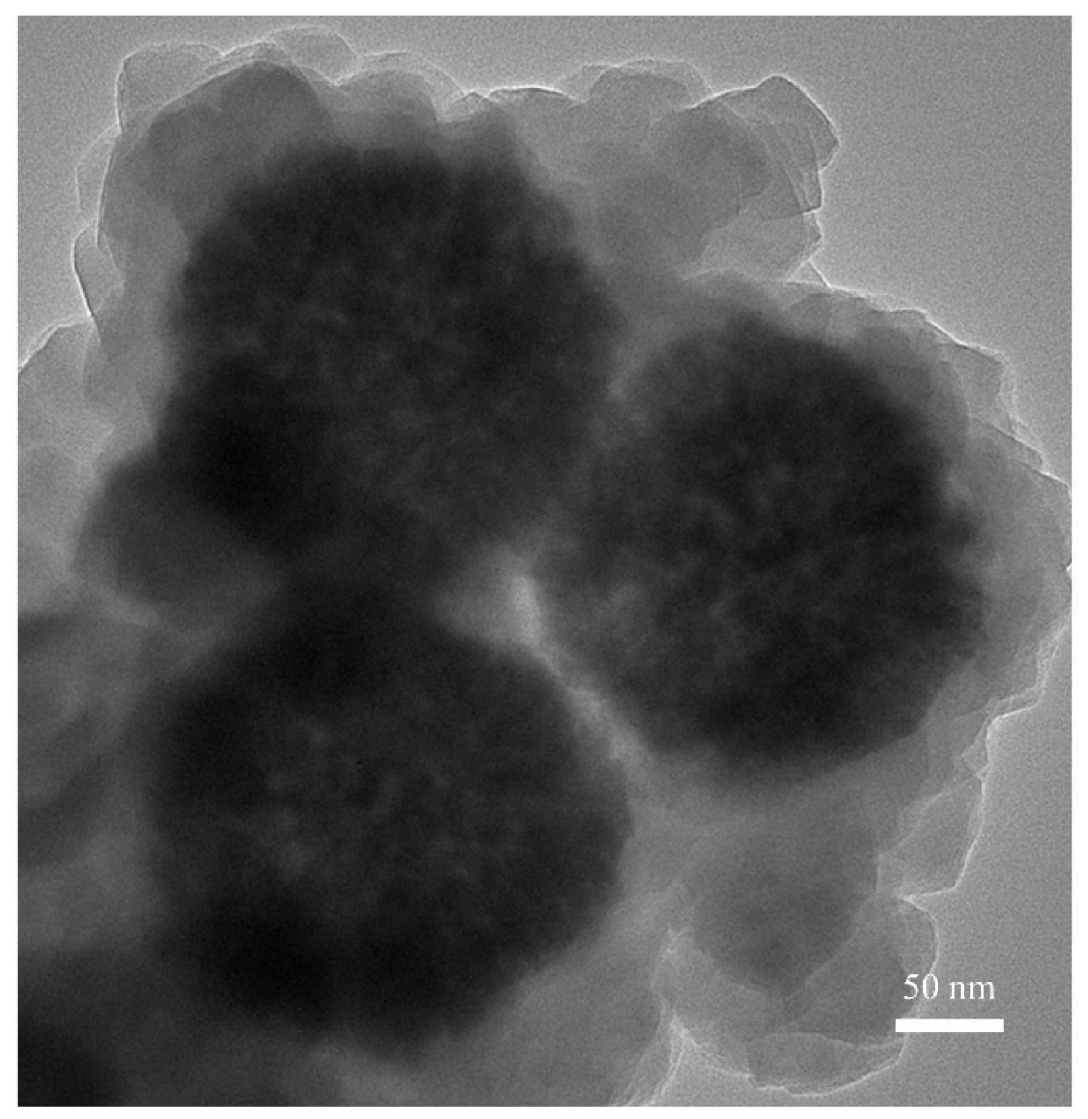
TEM images of Fe_3_O_4_@SiO_2_@UiO-66.

The morphology and dimensions of the synthesized material were studied by SEM and TEM. As shown in Figure 1a, Fe_3_O_4_ nanoparticles tend to be closely packed due to their small size and supermagnetism, which confirms that Fe_3_O_4_ nanoparticles can be used as a source of magnetic separation performance for the adsorbent. After silanization with TEOS, Fe_3_O_4_@SiO_2_ particles (Figure 1b) had a relatively smooth surface with a larger average size than untreated Fe_3_O_4_. After further compositing of UiO-66, the surface morphology of the material changed significantly, revealing tetrahedral and octahedral structures of UiO-66 and indicating the successful synthesis of the Fe_3_O_4_@SiO_2_@UiO-66 material. The strong Zr, Si, and Fe signals observed in the EDS mapping (Figure S1), as well as the core–shell structure of magnetic composite observed in the TEM image (Figure 2), also confirm the successful synthesis of Fe_3_O_4_@SiO_2_@UiO-66.

**Figure 3:**
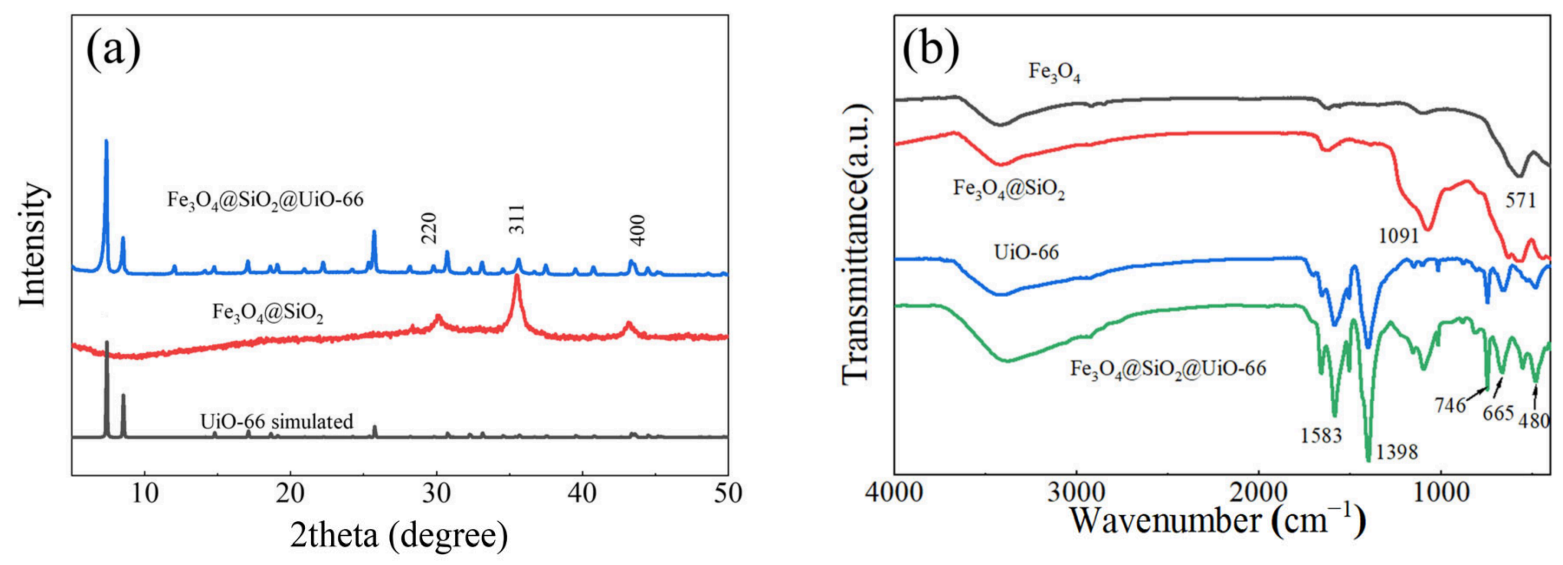
(**a**) XRD patterns of Fe_3_O_4_@SiO_2_, Fe_3_O_4_@SiO_2_@UiO-66, and UiO-66 simulated; (**b**) FTIR spectra of Fe_3_O_4_ and Fe_3_O_4_@SiO_2_, Fe_3_O_4_@SiO_2_@UiO-66, and UiO-66.

The crystal structures of Fe_3_O_4_@SiO_2_ and Fe_3_O_4_@SiO_2_@UiO-66 were investigated by using XRD. As presented in Figure 3a, the characteristic diffraction peaks of Fe_3_O_4_@SiO_2_ were in good agreement with those of 30.2, 35.5, and 43.3° of cubic crystalline magnetite (JCPDS card No.19-0629), which correspond to crystal planes (220), (311), and (400), respectively [54]. Fe_3_O_4_@SiO_2_@UiO-66 exhibited the peaks of Fe_3_O_4_@SiO_2_, indicating the composite material was magnetic. Moreover, the XRD patterns of Fe_3_O_4_@SiO_2_@UiO-66 showed characteristic diffraction peaks at 7.4°, 8.5°, 25.8°. These peaks are closely aligned with the diffraction patterns of the simulated UiO-66, signifying that the crystal structure of magnetic UiO-66 remained unaltered following its recombination with Fe_3_O_4_@SiO_2_. These observed features are consistent with the SEM image results.

In order to investigate the structure and functional groups of the sample, FTIR analysis was conducted. The spectra of UiO-66, Fe_3_O_4_, Fe_3_O_4_@SiO_2,_ and Fe_3_O_4_@SiO_2_@UiO-66 are shown in Figure 3b. There was a characteristic peak at 571 cm^−1^, which was related to Fe–O–Fe vibration within the lattice of Fe_3_O_4_. An obvious absorption band appeared at 1091 cm^−1^, corresponding to the stretching vibration of Si–O–Si [49]. The bands at 1583 and 1398 cm^−1^ are assigned to the asymmetric and symmetric stretching vibrations of the carboxylate (O = C–O) groups of the H_2_BDC linkers, respectively. There was a significant peak at 1506 cm^−1^, which was characteristic of C = C vibrations of the benzene ring [55]. In addition, the bands at 746 cm^−1^ and 665 cm^−1^ are attributed to the O-H and C-H vibrations in the H_2_BDC ligand, while the band at approximately 480 cm^−1^ is assigned to the Zr–O stretching vibrations of the Zr_6_ cluster, confirming the integrity of the UiO-66 framework in the composite [35,56,57]. The complete band assignments are summarized in Table S1 of the Supporting Information. Collectively, the FTIR analysis confirmed the successful coating of UiO-66 on Fe_3_O_4_@SiO_2_.

**Figure 4:**
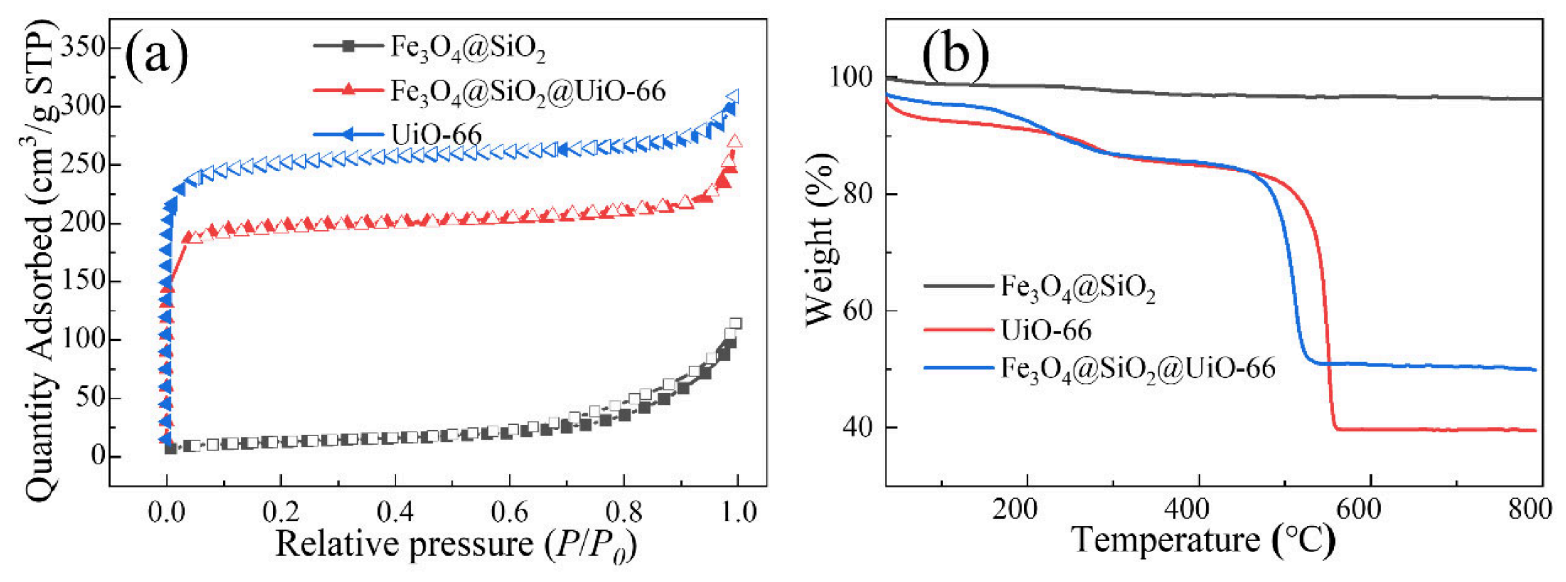
(**a**) N_2_ adsorption–desorption isotherms and (**b**) TGA curves of Fe_3_O_4_@SiO_2_, UiO-66, and Fe_3_O_4_@SiO_2_@UiO-66.

For a porous adsorbent, the adsorption performance was affected by porosity, pore size, and specific surface area, which were investigated through N_2_ adsorption–desorption experiment. As shown in Figure 4a and Table 1, the N_2_ adsorption–desorption isotherm indicated that UiO-66 and Fe_3_O_4_@SiO_2_@UiO-66 exhibited type I isotherms with H1 hysteresis loops, which proves their relatively regular microporous structure. Compared with UiO-66, the specific surface area of Fe_3_O_4_@SiO_2_@UiO-66 decreased from 759 m^2^/g to 622 m^2^/g due to the presence of the Fe_3_O_4_@SiO_2_ core, but it still could provide rich adsorption sites for the extraction of BA.

Thermal stability evaluations of Fe_3_O_4_@SiO_2_, UiO-66, and Fe_3_O_4_@SiO_2_@UiO-66 were conducted using TGA. The TGA curves in Figure 4b showed that the weight loss of Fe_3_O_4_@SiO_2_ was less than 4%, indicating that Fe_3_O_4_@SiO_2_ exhibited high thermal stability at temperatures up to 800 °C. For UiO-66 and Fe_3_O_4_@SiO_2_@UiO-66, they exhibited the same trend in TGA curves. The weight loss below 200 °C was mainly attributed to the removal of residual DMF and water in the channel. The gradual weight loss between 200 and 400 °C might be due to dehydroxylation of the MOF materials. When the temperature was raised from 400 °C to 800 °C, the organic ligands in UiO-66 decomposed, and the framework gradually collapsed and carbonized. Notably, the linker-decomposition step of Fe_3_O_4_@SiO_2_@UiO-66 begins at a slightly lower temperature than that of pristine UiO-66, which can be attributed to the thin MOF shell with small crystallites facilitating heat transfer, and to the catalytic effect of Fe_3_O_4_ on the oxidative decomposition of the linkers under an air atmosphere. The number of missing linkers per Zr_6_ cluster was calculated by comparing the measured ligand weight percentage with that of ideal UiO-66, following the reported method [58,59]. The detailed calculation procedure is provided in Section S1 of the Supporting Information. Through calculation, the number of missing linkers per Zr_6_ cluster in UiO-66 and Fe_3_O_4_@SiO_2_@UiO-66 was found to be 0.97 and 0.24, respectively. The addition of magnetic materials reduced defects in the crystal structure of UiO-66, which further indirectly confirmed the successful encapsulation of UiO-66 within Fe_3_O_4_@SiO_2_.

**Figure 5:**
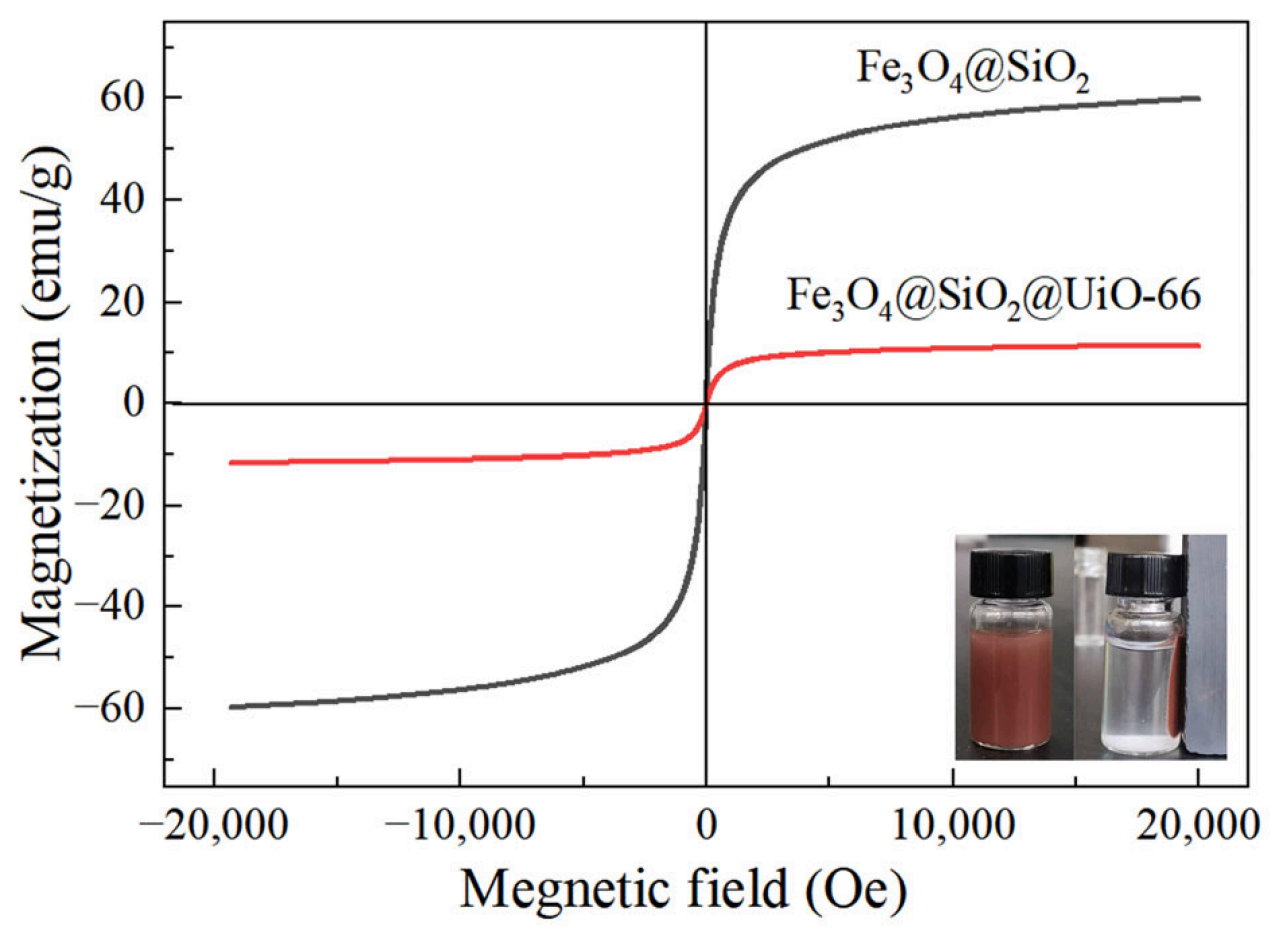
Magnetic hysteresis loops of Fe_3_O_4_@SiO_2_ and Fe_3_O_4_@SiO_2_@UiO-66.

In practical applications, magnetic properties assume a crucial role in liquid recovery and separation processes. As depicted in Figure 5, the hysteresis loops for both Fe_3_O_4_@SiO_2_ and Fe_3_O_4_@SiO_2_@UiO-66 were examined. It was observed that Fe_3_O_4_@SiO_2_ exhibited a saturation magnetization of 59.9 emu/g, whereas Fe_3_O_4_@SiO_2_@UiO-66 displayed a somewhat reduced saturation magnetization of 11.5 emu/g. This reduction in magnetization can be attributed to the presence of a substantial UiO-66 coating on Fe_3_O_4_@SiO_2_. Nonetheless, the resulting magnetic UiO-66 composite retained a sufficient level of magnetic responsiveness, enabling efficient separation under the influence of an external magnetic field. As illustrated in the inset of Figure 5, the adsorbent could be effortlessly and fully separated when subjected to an external magnetic field.

### 3.2. Adsorption Kinetics

Adsorption kinetics is an important means of evaluating the performance of adsorbents. Under the conditions of 30 °C and an initial concentration of 1000 mg/L, the effect of different contact times on the adsorption of benzoic acid by UiO-66 and Fe_3_O_4_@SiO_2_@UiO-66 is shown in Figure S2. Based on the results obtained from ICP (Table S2) and TGA analyses, the adsorption capacity per Zr_6_ cluster unit in UiO-66 was determined to be 450.38 g/mol, whereas in Fe_3_O_4_@SiO_2_@UiO-66, this value increased significantly to be 569.47 g/mol, marking a notable enhancement of over 26% compared to UiO-66 in isolation. These findings strongly indicate the potential existence of a synergistic effect between magnetic particles and UiO-66, thereby leading to the augmentation of benzoic acid adsorption. For comparison, Fe_3_O_4_@SiO_2_ alone was also tested under identical conditions (30 °C, initial BA concentration of 1000 mg/L, adsorbent dosage of 1 g/L, 4 h). It exhibited a much lower BA adsorption capacity (17.25 mg·g^−1^, Figure S2), confirming that the adsorption capacity of the composite arises predominantly from the UiO-66 shell, while the magnetic core contributes the magnetic separation and templating functions.

**Figure 6:**
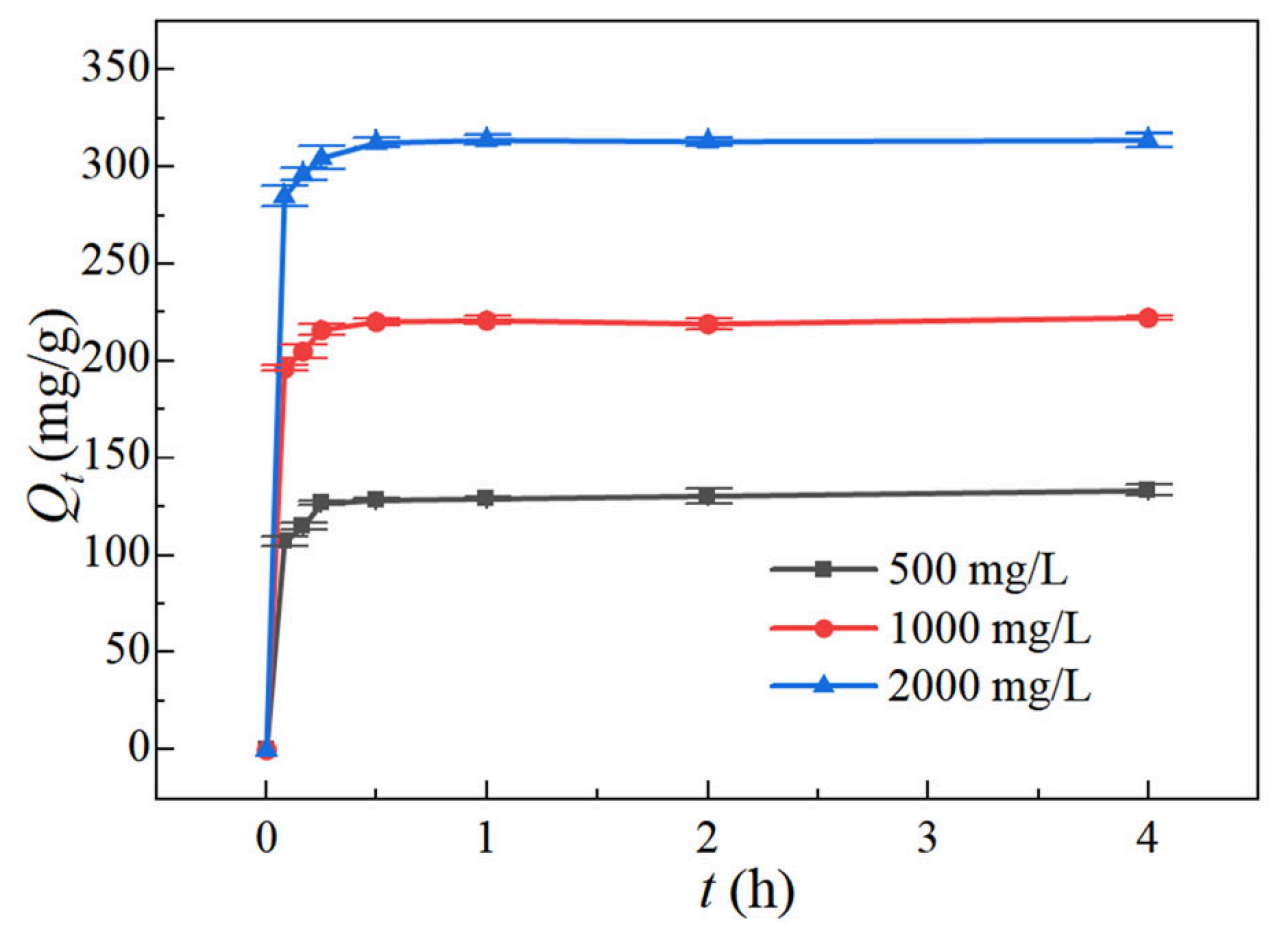
Adsorption kinetics of BA on Fe_3_O_4_@SiO_2_@UiO-66.

For Fe_3_O_4_@SiO_2_@UiO-66, a rapid increase in adsorption capacity was observed within half an hour, and equilibrium was reached in one hour (Figure 6). At the early stage of the adsorption process, Fe_3_O_4_@SiO_2_@UiO-66 had a faster adsorption rate for BA molecules, which could be because of the large concentration difference between Fe_3_O_4_@SiO_2_@UiO-66 and the solution, and the existence of a large number of adsorption vacancies in the adsorbent material. With the progress of adsorption, the number of remaining adsorption active sites of Fe_3_O_4_@SiO_2_@UiO-66 gradually decreased, which caused a gradual decrease in the driving force [60]. As a result, the adsorption amount slowly increased until the adsorption equilibrium was achieved. By comparing BA adsorption at different initial concentrations, it was found that the adsorption capacity of BA on Fe_3_O_4_@SiO_2_@UiO-66 increased with the increase in initial concentration of BA. Therefore, magnetic UiO-66 was more suitable for adsorbing high-concentration BA aqueous solution, which was of great significance for the industrial application of the adsorbent. The normalized concentration profiles (C/C_0_) versus time (Figure S3) show that C/C_0_ levels off at 0.74, 0.78, and 0.84 for initial BA concentrations of 500, 1000, and 2000 mg·L^−1^, respectively. This confirms that complete removal of BA is not achieved at the plateau and that adsorption equilibrium is established with BA molecules distributed between the adsorbent and the aqueous solution.

**Figure 7:**
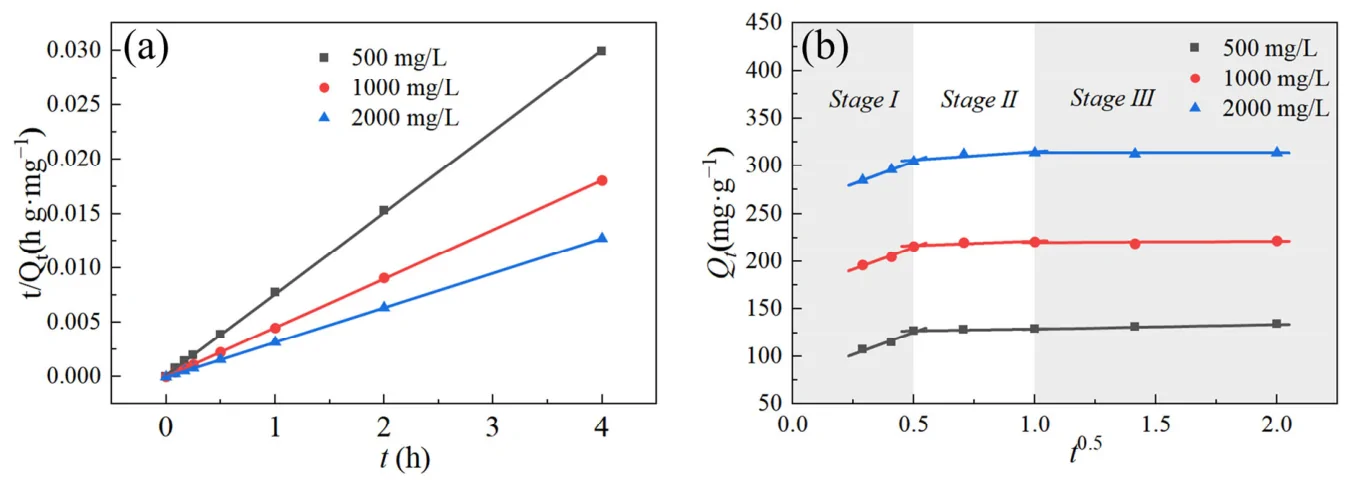
The (**a**) pseudo-second-order kinetics plots and (**b**) intraparticle diffusion kinetics plots fittings of BA adsorption on Fe_3_O_4_@SiO_2_@UiO-66.

To investigate the adsorption kinetics of benzoic acid on Fe_3_O_4_@SiO_2_@UiO-66, a pseudo-second-order kinetic model and an intraparticle diffusion model were adopted. The linear fitting curves are shown in Figure 7, and the relevant parameters and correlation coefficients for both kinetic models were provided in Table 2. Notably, the correlation coefficients for the pseudo-second-order model were consistently above 0.999, indicating an excellent fit to the kinetic data, and the equilibrium adsorption capacities closely aligned with experimental results. Consequently, it can be inferred that the adsorption process primarily involves chemical adsorption, characterized by electron sharing or exchange between benzoic acid (BA) molecules and the adsorbent. When the kinetic data were fitted with the intraparticle diffusion model, the plots of Q_t_ versus t^0.5^ were multi-linear and could be divided into three stages (Figure 7b; Table 2), indicating that BA adsorption proceeds through consecutive diffusion steps. The first and steepest stage (Stage I) corresponds to the transfer of BA molecules from the bulk solution across the boundary layer to the external surface of the adsorbent; the non-zero intercepts (*K*_1_ = 79.0–258.0 mg·g^−1^) indicate that the plots do not pass through the origin and that intraparticle diffusion is not the sole rate-controlling step. The second stage (Stage II) is attributed to the gradual diffusion of BA molecules into the micropores of the UiO-66 shell, with rate constants *k_i_*_2_ (4.26–17.71 mg·g^−1^·h^−0.5^) much smaller than those of Stage I (*k_i_*_1_ = 92.4–93.3 mg·g^−1^·h^−0.5^). The third stage (Stage III) corresponds to the final adsorption–desorption equilibrium, during which the residual BA concentration and the available surface sites become depleted; its rate constant approaches zero at higher initial concentrations (*k_i_*_3_ = 1.40 and 0.18 mg·g^−1^·h^−0.5^ for 1000 and 2000 mg·L^−1^, respectively). Overall, the rate constant for Stage I is one to two orders of magnitude larger than those of the subsequent stages, confirming the consecutive three-stage mechanism. It should be noted that, because adsorption equilibrium is reached rapidly (within ca. 1 h), the second and third stages are not always clearly separated; at the lowest initial concentration, their slopes are even nearly identical. This observation suggests that the rate-limiting step in the process may be associated with the migration and diffusion of BA within the framework [50].

### 3.3. Adsorption Isotherms

**Figure 8:**
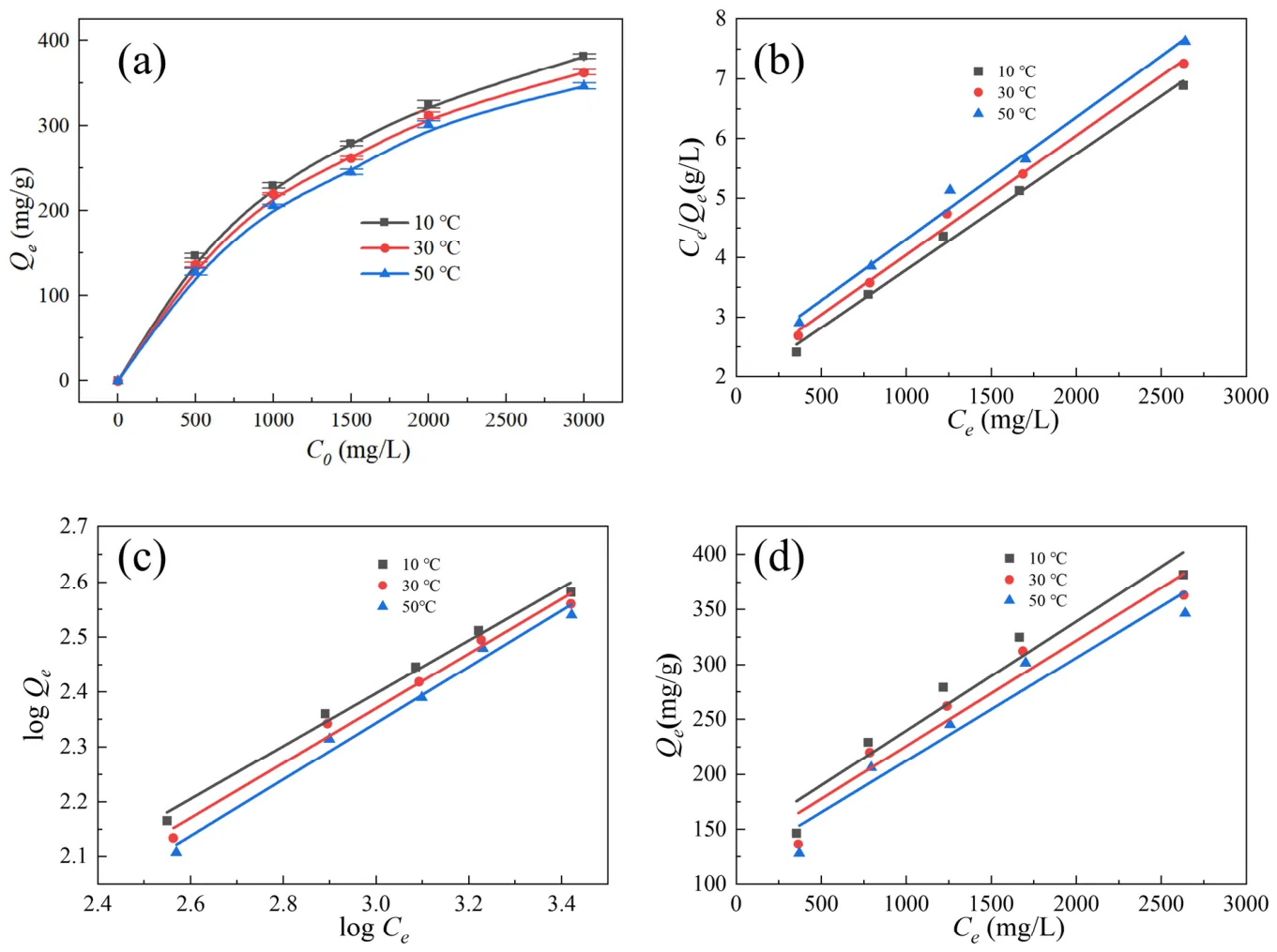
(**a**) Adsorption isotherms of BA on Fe_3_O_4_@SiO_2_@UiO-66 fitted by using (**b**) the Langmuir model, (**c**) the Freundlich model, and (**d**) the Henry model.

The adsorption isotherms could also provide valuable information for understanding adsorption behavior. The adsorption isotherms of BA on Fe_3_O_4_@SiO_2_@UiO-66 were determined at 10, 30, and 50 °C for a duration of 4 h, as presented in Figure 8a. As the initial BA concentration increased, the BA adsorption amount increased, whereas its adsorption amount decreased with increasing temperature, which showed that adsorption of BA on Fe_3_O_4_@SiO_2_@UiO-66 was an exothermic process.

As shown in Figure 8b–d, linear fitting curves based on isotherm models were presented. Table 3 summarizes the correlation coefficients (*R*^2^) and the isotherm parameters obtained. As compared to the Freundlich and Henry isotherm models, Langmuir isotherms could better describe the adsorption isotherms of BA on Fe_3_O_4_@SiO_2_@UiO-66 because of their higher R^2^. The theoretical maximum capacity calculated by the Langmuir model at 10 °C is 512.82 mg/g, which is much higher than reported in previous studies, as shown in Table S3 [4,16,61,62,63]. Therefore, magnetic UiO-66 showed superior adsorption performance and could be easily separated from the solution in the presence of external magnetic fields rather than complex filtration procedures, making it more attractive for practical applications.

### 3.4. Adsorption Thermodynamics

**Figure 9:**
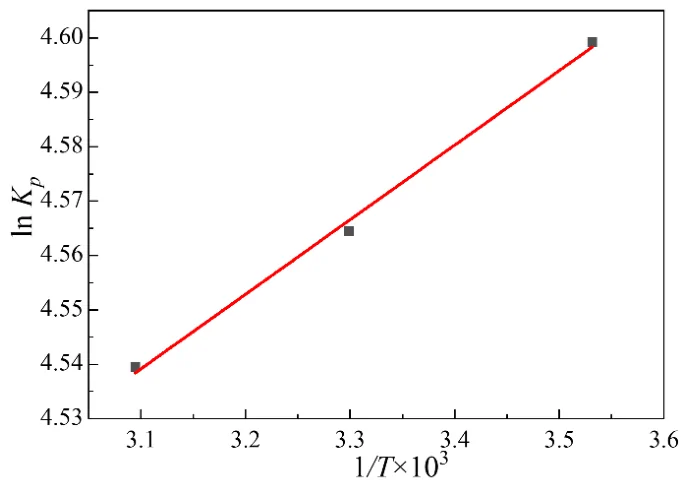
Van ’t Hoff plot.

Based on the thermodynamic calculation (Figure 9, Table 4), the spontaneous BA adsorption process on magnetic UiO-66 could be confirmed by the negative Δ*G*, while the negative Δ*H* (−1.14 kJ·mol^−1^) further demonstrated the existence of exothermic adsorption in the process of BA adsorption, which was consistent with the fact that the adsorption amount of BA increased with decreasing temperature. While the Δ*S* (0.0342 kJ·mol^−1^·K^−1^) indicated an increase in randomness of the adsorbent–adsorbate interface.

### 3.5. Adsorption Mechanism

**Figure 10:**
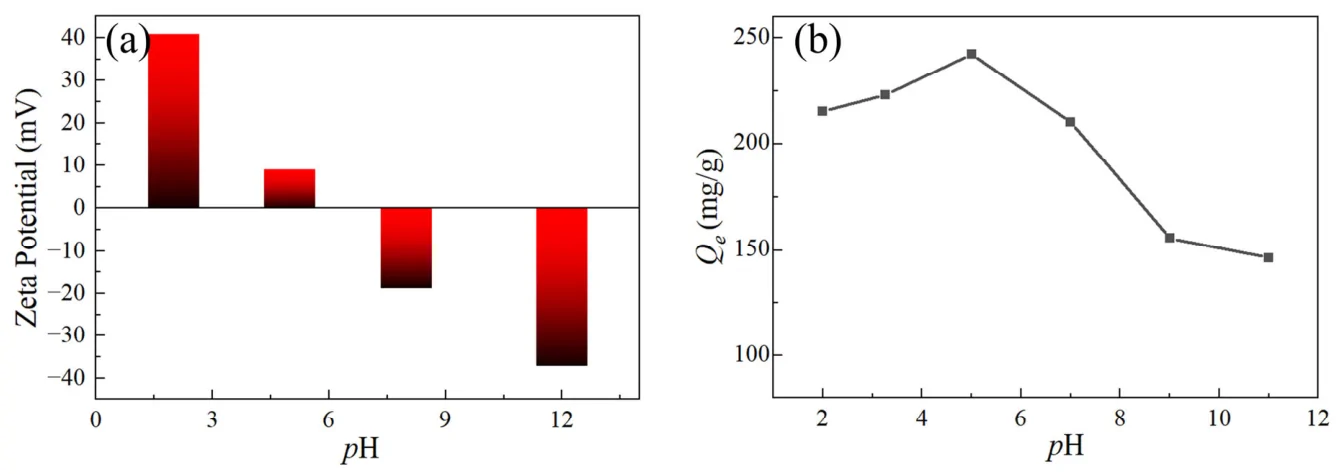
(**a**) The zeta potential of Fe_3_O_4_@SiO_2_@UiO-66 at corresponding pH values; (**b**) effect of solution pH on the adsorption of BA on Fe_3_O_4_@SiO_2_@UiO-66.

In order to design adsorbents with high efficiency and large capacity, it was indispensable to explore the adsorption mechanism and understand the interaction between adsorbents and target molecules. The initial pH applied has an impact on both the surface charge of the adsorbents and the state of existence of the target adsorbates. Therefore, at corresponding pH values, we determined Fe_3_O_4_@SiO_2_@UiO-66 zeta potentials and investigated the effects of pH on adsorption amount (Figure 10). The adsorption amount of BA on Fe_3_O_4_@SiO_2_@UiO-66 increased with rising pH up to about 5 and then gradually decreased with the continued increasing pH value. The zeta potential of magnetic UiO-66 materials could be applied to explain this tendency of BA adsorption. As shown in Figure 10a, the zero charge point of Fe_3_O_4_@SiO_2_@UiO-66 was approximately at pH = 5.5, which meant that the surface charge of magnetic UiO-66 remained positive when pH is lower than 5.5 and became negative when pH is higher than 5.5. BA is a weak acid with the pK_a_ value of 4.20 at 298 K; thus, BA molecules exist mainly as neutral BA molecules at pH less than 4.2, while BA molecules deprotonate into negatively charged ions at pH greater than 4.2 [64]. Therefore, when the pH was below 5, the negatively charged BA molecule might interact with the positively charged Fe_3_O_4_@SiO_2_@UiO-66 surface through electrostatic interaction. When the pH value was greater than 5 (especially above 5.5), the adsorption capacity began to decline, probably due to the electrostatic repulsion of negatively charged BA molecules and negatively charged Fe_3_O_4_@SiO_2_@UiO-66 surface.

In the concentration range we studied (500–2000 mg/L), the pH value of the solution was less than 4.2, and only a small part of BA could be dissociated in the form of ions, with neutral molecules being the dominant form. However, Fe_3_O_4_@SiO_2_@UiO-66 still exhibited excellent adsorption performance for the BA molecule, suggesting that there might be other factors that dominate the adsorption. Some previous studies have reported that the adsorption of the neutral BA molecule onto functionalized carbon nanotubes was partially because of π–π stacking interactions [65]. Since the imidazole ring was similar to the bond and electron distribution of the benzene ring, the 2-methylimidazole molecule was considered to be an aromatic molecule, and π–π stacking interactions existed between the ZIF-8 composite and aromatic BA molecules [4]. Similarly, terephthalic acid, the ligand of UiO-66, could also interact with BA molecules via the π–π stacking interaction, thus enhancing adsorption capability for BA.

**Figure 11:**
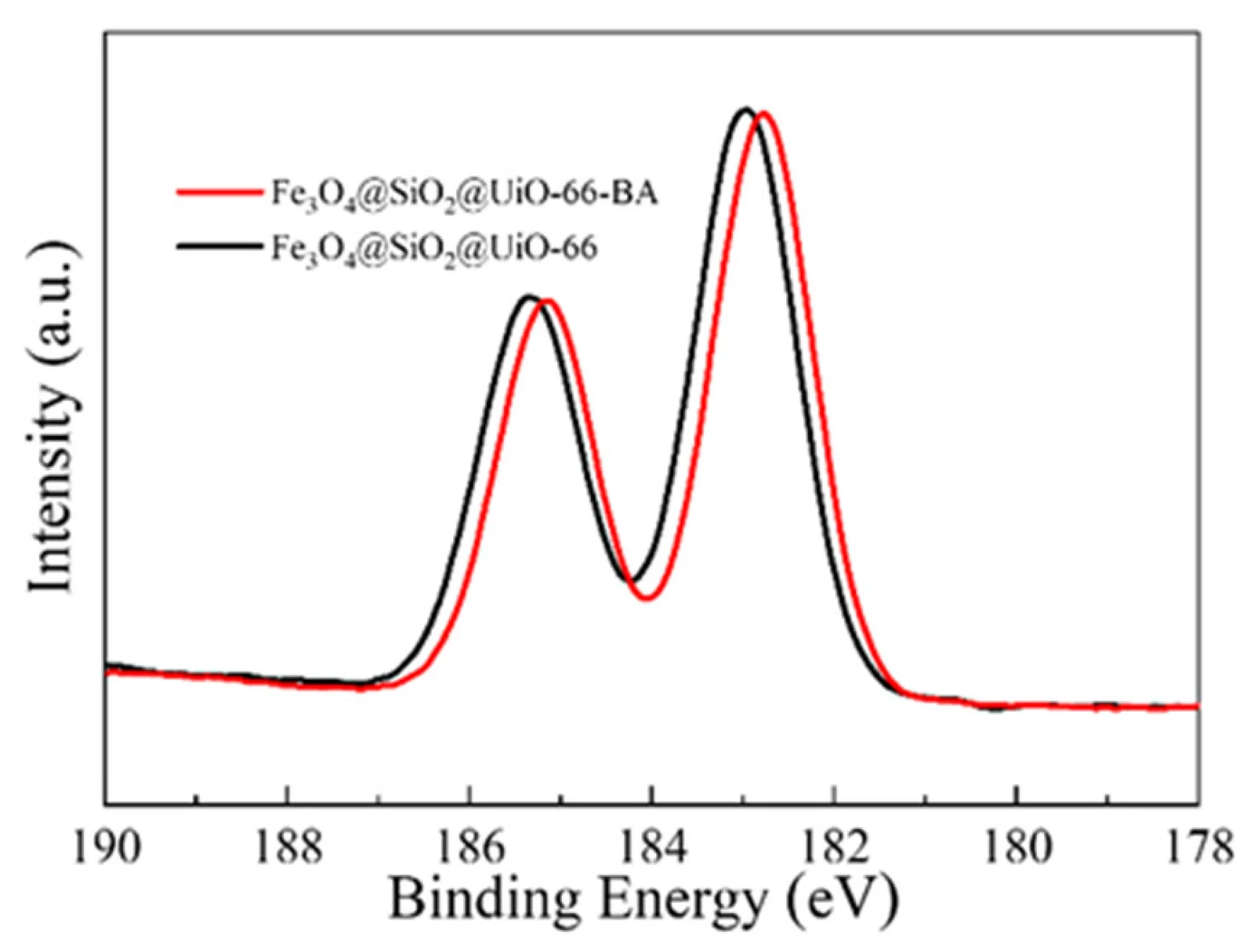
Zr3d XPS spectra of pristine and BA adsorbed Fe_3_O_4_@SiO_2_@UiO-66.

In order to investigate the presence of coordination interactions, XPS analysis was applied to observe the alteration in the binding energies of elements on the surface of the adsorbent before and after adsorption. As evident from Zr3d XPS spectra (Figure 11), the peaks of Zr3d moved slightly to lower binding energy after adsorption, which indicated that coordination interactions occurred in the BA adsorption process. Based on the above discussion, it could be concluded that the adsorption of BA over Fe_3_O_4_@SiO_2_@UiO-66 was caused by the synergistic effects of different mechanisms, including electrostatic interactions, coordination interactions, and π–π stacking interactions.

### 3.6. Regeneration and Reusability

**Figure 12:**
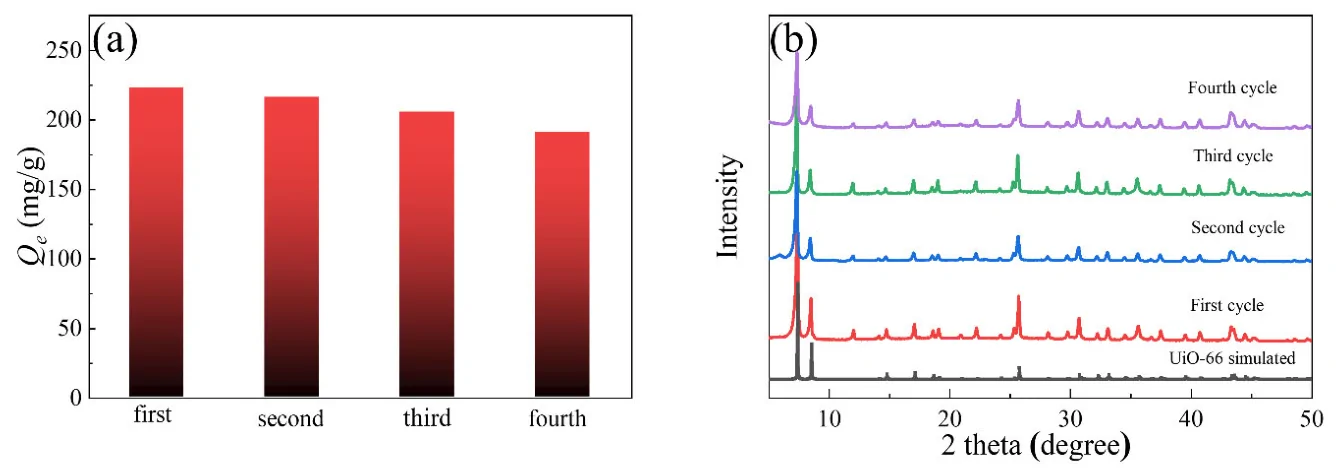
(**a**) Four-cycle BA adsorption over Fe_3_O_4_@SiO_2_@UiO-66; (**b**) XRD patterns of Fe_3_O_4_@SiO_2_@UiO-66 after each cycle in four adsorption−desorption processes.

The regeneration of the adsorbent must be considered in practical applications. As shown in Figure 12a, after a four-cycle adsorption and desorption experiment, the adsorption capacities of BA over magnetic UiO-66 did not decrease significantly, and the regeneration efficiency still remained over 85%, which showed that the adsorbent exhibited excellent reusability. Furthermore, XRD analysis (Figure 12b) confirmed that the crystal structure of Fe_3_O_4_@SiO_2_@UiO-66 remained well-preserved, indicating its substantial stability. After four cycles of adsorption and regeneration, the saturation magnetization of Fe_3_O_4_@SiO_2_@UiO-66 was 10.8 emu/g, which meant that the magnetic property of the adsorbent was virtually unchanged compared with that before adsorption (Figure S4).

## 4. Conclusions

In this paper, we successfully synthesized a magnetic UiO-66 composite and employed it as an efficient adsorbent for the removal of BA from water. The observed potential synergy between magnetic particles and UiO-66 contributed to improved benzoic acid adsorption performance. The adsorption capacity per Zr_6_ cluster unit in Fe_3_O_4_@SiO_2_@UiO-66 reached 569.5 g/mol, representing a remarkable enhancement of over 26% compared to UiO-66. Adsorption kinetics and isotherm analyses indicated that the BA adsorption process primarily involved chemical adsorption occurring on the homogeneous surface of the adsorbent, with a calculated theoretical adsorption capacity reaching an impressive 512.8 mg/g. Adsorption thermodynamics studies revealed that the adsorption of BA over Fe_3_O_4_@SiO_2_@UiO-66 was spontaneous and exothermic. We elucidated the possible mechanisms governing BA adsorption, emphasizing the roles of electrostatic interactions, coordination interactions, and π–π stacking interactions. Regeneration studies showed that the regeneration efficiency of Fe_3_O_4_@SiO_2_@UiO-66 remained over 85% even after multiple reuse cycles and maintained good structural stability and magnetic separation capabilities. Therefore, these findings could provide valuable strategies for the development of efficient, stable, and easily recoverable MOF-based adsorbents for BA removal in water.

## Figures and Tables

**Table 1:** Textural properties of Fe_3_O_4_@SiO_2_, UiO-66, and Fe_3_O_4_@SiO_2_@UiO-66.

Material	BET Surface Area (m^2^/g)	Total Pore Volume (cm^3^/g)
Fe_3_O_4_@SiO_2_	46	0.16
UiO-66	759	0.43
Fe_3_O_4_@SiO_2_@UiO-66	622	0.40

**Table 2:** BA adsorption kinetic parameters of the pseudo-second-order and the intraparticle diffusion model. The k_i_, K, and R^2^ values of the intraparticle diffusion model are listed in the order of Stage I, Stage II, and Stage III (from top to bottom).

Adsorbent	*Q_e_* (exp.) (mg·g^−1^)	*Q_e_* (cal.) (mg·g^−1^)	*k*_2_ (g·mg^−1^·h^−1^)	*R* ^2^	*K* (mg·g^−1^)	*k_i_* (mg·g^−1^·h^−0.5^)	*R* ^2^
500	136.3	136.4	0.1920	0.9999	79.01 125.0 124.30	93.28 4.261 4.730	0.9641 0.9220 0.9989
1000	223.1	223.2	0.3410	0.9999	168.92 212.1 218.66	92.38 9.457 1.399	0.9774 0.8293 0.1957
2000	313.3	313.5	0.8855	1.000	257.97 297.1 313.18	93.01 17.71 0.184	1.0000 0.7904 0.0277

**Table 3:** The parameters of three isotherm models for BA adsorption over Fe_3_O_4_@SiO_2_@UiO-66.

T (°C)	Langmuir Model			Freundlich Model			Henry Model	
	*Q_m_* (mg·g^−1^)	*K_A_* (L·g^−1^)	*R* ^2^	1/*n*	*K_F_*	*R* ^2^	*K_p_* (mL·g^−1^)	*R* ^2^
10	512.8	1.056	0.9951	0.4802	9.056	0.9884	99.41	0.9149
30	497.5	0.9864	0.9939	0.4976	7.537	0.9824	96.01	0.9108
50	487.8	0.9093	0.9885	0.5123	6.402	0.9829	93.64	0.9152

**Table 4:** Thermodynamic parameters of BA adsorption over Fe_3_O_4_@SiO_2_@UiO-66.

*T* (°C)	Δ*G* kJ·mol^−1^	Δ*S* kJ·mol^−1^·K^−1^	Δ*H* kJ·mol^−1^
10	−10.8	0.0342	−1.14
30	−11.5		
50	−12.2		

## Data Availability

The data are contained within the article.

## Supplementary Materials

The following supporting information can be downloaded at: https://www.mdpi.com/article/10.3390/molecules31162759/s1. Section S1. Calculation of the number of missing linkers per Zr_6_ cluster; Figure S1. EDX mappings of the portion selected: distribution of O (a), Zr (b), Si (c), and Fe (d); Figure S2. Adsorption performance of BA on Fe_3_O_4_@SiO_2_, UiO-66 and Fe_3_O_4_@SiO_2_@UiO-66 (temperature = 30 °C, initial concentration = 1000 mg/L); Figure S3. Normalized concentration (C/C_0_) versus time for BA adsorption on Fe_3_O_4_@SiO_2_@UiO-66; Figure S4. Magnetization curves of Fe_3_O_4_@SiO_2_@UiO-66 after four cycles of adsorption and regeneration. Table S1. FTIR band assignments of Fe_3_O_4_@SiO2@UiO-66; Table S2. ICP data of Fe_3_O_4_@SiO_2_@UiO-66 sample; Table S3. Comparison of BA adsorption capacities for Fe_3_O_4_@SiO_2_@UiO-66 with those of other adsorbents.
