# Supplementary material for: Synergistic Adsorption by Easily Retrievable Magnetic MOF Composites for Enhanced Removal of Benzoic Acid from Water
Source: Molecules. 2026 Aug 8;31(16):2759. doi: 10.3390/molecules31162759 (PMC13515108; doi:10.3390/molecules31162759)
Supplement: Supplementary file 1 [file molecules-31-02759-s001.zip › molecules-4438687-supplementary.pdf]

Supporting Information

Synergistic Adsorption by Easily Retrievable Magnetic MOF Composites for Enhanced Removal of Benzoic Acid from Water

Panpan Liu [1,*], Peijun He [2], Yixin Wu [2], Xiufang Li [1,*], Xianmang Xu [2], Jianing Duan [2] and Peng Bai [3,4]

[1] College of Pharmacy, Heze University, Heze 274015, China
[2] Heze Branch, Qilu University of Technology (Shandong Academy of Sciences), Heze 274000, China; peijun_he2026@163.com (P.H.); wuyixin0105@163.com (Y.W.); xuxianmang168@qlu.edu.cn (X.X.); djn@qlu.edu.cn (J.D.)
[3] Department of Pharmaceutical Engineering, School of Chemical Engineering and Technology, Tianjin University, Tianjin 300350, China; baipenga525@tju.edu.cn
[4] Key Laboratory of Systems Bioengineering, Ministry of Education, Tianjin 300350, China
* Correspondence: panpan_liu@126.com (P.L.); lixiufang_2030@163.com (X.L.)

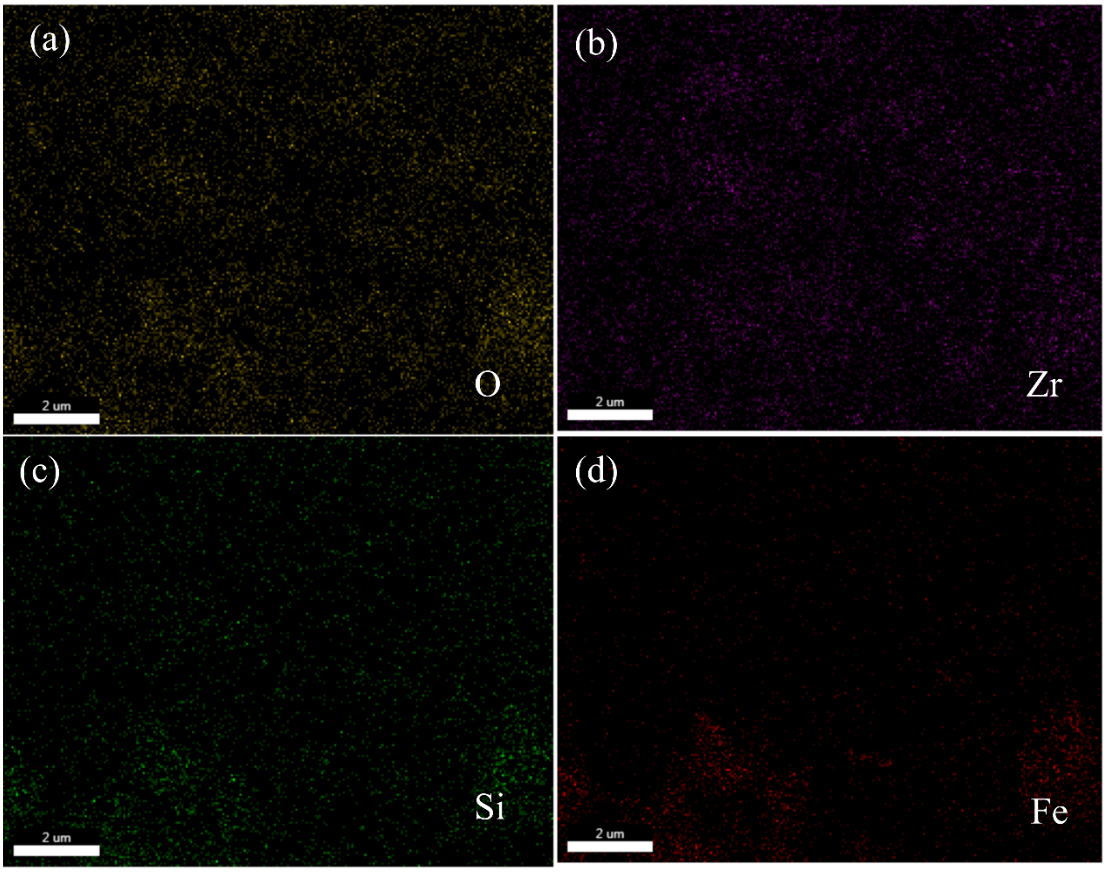

Fig. S1. EDX mappings of the portion selected: distribution of O (a), Zr (b), Si (c) and Fe (d).

Table S1. FTIR band assignments of Fe_3O_4@SiO_2@UiO-66

Wavenumber (cm^{-1})	Assignment
1583	Asymmetric stretching of carboxylate (O=C-O) of the BDC linkers
1506	C=C stretching of the benzene ring of the BDC linkers
1398	Symmetric stretching of carboxylate (O=C-O) of the BDC linkers
1091	Asymmetric stretching of Si-O-Si
746	O-H vibration in the H_2BDC ligand
665	C-H vibration of the terephthalate benzene ring
571	Fe-O-Fe vibration of the Fe_3O_4 lattice
480	Zr-O vibration of the Zr_6 cluster

Table S2. ICP data of Fe3O4@SiO2@UiO-66 sample

Fe content wt%	Si content wt%	Zr content wt%	Fe:Si:Zr molar ratio in the framework

14.08	5.056	18.01	7.66:5.47:6

Section S1. Calculation of the number of missing linkers per Zr_6 cluster

For ideal, defect-free UiO-66, each Zr_6 cluster carries 6 BDC linkers, giving the composition $Zr_6O_6(BDC)_6$ (M = 1628.03 g·mol^{-1}). Upon heating in air, the framework is completely combusted and each Zr_6 cluster is converted into 6 ZrO_2 (739.34 g·mol^{-1}) ($Zr_6O_6(BDC)_6 + 45\ O_2 \rightarrow 6\ ZrO_2 + 48\ CO_2 + 12\ H_2O$). The ligand weight percentage of the ideal framework is therefore (1628.03 − 739.34)/1628.03 = 54.6%. For a defective sample, formulated as $Zr_6O_{6+x}(BDC)_{6-x}$, each missing linker is charge-compensated by an additional oxide anion on the Zr_6 cluster, and the ligand weight percentage decreases accordingly; the number of missing linkers per Zr_6 cluster can thus be obtained by comparing the measured ligand weight percentage with the ideal value. For UiO-66, the weight loss ascribed to the combustion of the BDC linkers accounts for 45.8% of the desolvated material, which is lower than the ideal value of 54.6%, and the number of missing linkers per Zr_6 cluster was calculated to be 0.97.

For Fe_3O_4@SiO_2@UiO-66, the inorganic residue after combustion contains not only ZrO_2 but also the iron and silicon oxides originating from the Fe_3O_4@SiO_2 core, which is thermally stable up to 800 °C (<4% weight change, Fig. 4b). The fraction of ZrO_2 in the total inorganic residue (44%) was determined from the ICP results (Table S2, Fe:Si:Zr = 7.66:5.47:6, with Fe and Si converted to Fe_2O_3 and SiO_2 under the air atmosphere). Based on this, the ligand weight percentage relative to the UiO-66 portion of the composite was determined, from which the number of missing linkers per Zr_6 cluster in the composite was calculated to be 0.24. The lower defect concentration of the composite compared with pristine UiO-66 indicates that the introduction of the magnetic core reduces the defects in the crystal structure of UiO-66.

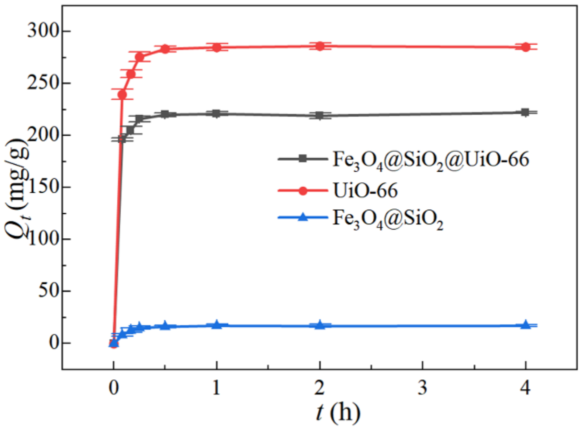

Fig. S2. Adsorption performance of BA on Fe_3O_4@SiO_2, UiO-66 and Fe_3O_4@SiO_2@UiO-66 (temperature = 30℃, initial concentration = 1000mg/L).

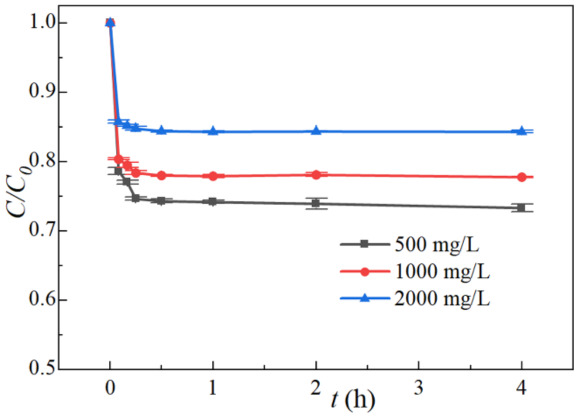

Fig. S3. Normalized concentration (C/C_0) versus time for BA adsorption on Fe_3O_4@SiO_2@UiO-66.

Table S3. Comparison of BA adsorption capacities for Fe3O4@SiO2@UiO-66 with those of other adsorbents

Adsorbent	Experimental conditions				Refs
	T (K)	Q_e(mg/g)	C_0(mg/L)	pH	
CTAB-montmorillonite	298	~38	1220	9	1
OTMAC-Bent	298	12.89	610	3.5	2
Al(OH)-OTMAC-Bent	298	13.28	610	3.5	2
Modified chitosan	298	170.08	2000	3.68	3
OTMAB modified bentonite	313	43.3	600	3	4
ZIF-8/MWNTs-25 (B)	303	303	500	~	5
UiO-66	303	286.35	1000	3.26	This study
Fe_3O_4@SiO_2@UiO-66	303	219.92	1000	3.26	This study
Fe_3O_4@SiO_2@UiO-66	283	512.82	~	~	This study

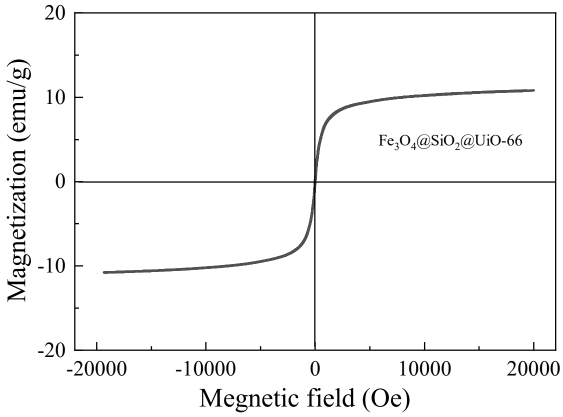

Fig. S4 Magnetization curves of Fe_3O_4@SiO_2@UiO-66 after four cycles of adsorption and regeneration.

References

[1] Yan L-G, Wang J, Yu H-Q, et al. Adsorption of benzoic acid by CTAB exchanged montmorillonite[J]. Applied Clay Science, 2007, 37(3): 226-230.

[2] Xin X, Si W, Yao Z, et al. Adsorption of benzoic acid from aqueous solution by three kinds of modified bentonites[J]. Journal of Colloid and Interface Science, 2011, 359(2): 499-504.

[3] Chai K, Ji H. Dual functional adsorption of benzoic acid from wastewater by biological-

based chitosan grafted β-cyclodextrin[J]. Chemical Engineering Journal, 2012, 203: 309-318
[4] N. Yıldız, R. Go¨nu¨ls¸ en, H. Koyuncu, A. C¸alımlı, Adsorption of benzoic acid and hydroquinone by organically modified bentonites[J]. Colloid Surf. Physiochem. Eng. Aspect., 2005, 260 : 87–94.
[5] Dai J, Xiao X, Duan S, et al. Synthesis of novel microporous nanocomposites of ZIF-8 on multiwalled carbon nanotubes for adsorptive removing benzoic acid from water[J]. Chem. Eng. J., 2018, 331: 64-74.
